# Extensive hypothesis testing for estimation of crash frequency models

**DOI:** 10.1016/j.heliyon.2024.e26634

**Published:** 2024-02-23

**Authors:** Zeke Ahern, Paul Corry, Wahi Rabbani, Alexander Paz

**Affiliations:** a School of Civil & Environment Engineering, Queensland University of Technology, 2 George Street, Brisbane, 4000 QLD, Australia; b School of Mathematical Sciences, Queensland University of Technology, 2 George Street, Brisbane, 4000 QLD, Australia; c Queensland Department of Transport and Main Roads, Brisbane, 4000 QLD, Australia

**Keywords:** Metaheuristic, Regression, Random parameters, Hypothesis testing, Crash data, Data count models, Optimization

## Abstract

Estimating crash data count models poses a significant challenge which requires extensive knowledge, experience, and meticulous hypothesis testing to capture underlying trends. Simultaneous consideration of multiple modelling aspects is required including, among others, functional forms, likely contributing factors, and unobserved heterogeneity. However, model development, frequently affected by time and knowledge, can easily overlook crucial modelling aspects such as identification of likely contributing factors, necessary transformations, and distributional assumptions. To facilitate model development and an estimation that can extract as many insights as possible, an optimization framework is proposed to generate and simultaneously test a diverse array of hypothesis. The framework comprises a mathematical programming formulation and three alternative solution algorithms. The objective function involves minimizing the Bayesian Information Criterion (BIC) to avoid overfitting. The solution algorithms include metaheuristics to deal with an NP-hard problem and search through a complex and nonconvex space. The metaheuristics also enable to handle unique datasets through varying search strategies. The effectiveness of the proposed framework was ascertained using three distinct datasets, and published models used as benchmarks. The results highlighted the ability of the proposed framework to estimate crash data count models, surpassing benchmark models in terms of insights and goodness-of-fit. The framework provides several advantages, such as robust hypothesis testing, uncovering unique specifications and vital insights in the data, and leveraging existing knowledge to enhance search efficiency. The framework also exposes the vulnerability of traditional analyst efforts to fall into local optima, bias, and limitations in creating more efficient models. In a compelling example using crash data from Washington, the proposed framework unveiled insights overlooked by a benchmark published model, identifying speed, interchanges, and grade breaks as likely crash contributors, and revealing the potential danger of excessively wide shoulders. Conversely, the benchmark model identified fewer contributing factors and missed a crucial non-linear relationship between crash safety and shoulder widths. While wider shoulders are typically associated with improved safety, the proposed models suggest a safety threshold beyond which further widening could decrease safety. The introduction of random parameters in the analysis revealed a more nuanced relationship with crash frequency, thereby underlining the limitations of models incapable of capturing heterogeneity.

## Highlights

• Formulation for guided testing in count models, considers selection of random parameters, distributions and transformations.
• Optimization framework with 3 metaheuristics for generating and testing specifications using simulated log-likelihood.
• Real data experiments show advantages of our framework. Lower development costs, similar insights, and capturing causality.
• Synthetic data experiments evaluate proposed approach, benchmarking against known synthetically generated model.
• Treating multiple estimation aspects as decision variables overcomes analyst limitations, minimizing bias and saving time.

## 1 Introduction

Crash severity and frequency models play a pivotal role in identifying potential contributing factors, thereby enabling the development of sound countermeasures [46], [96]. Despite the burgeoning progress in machine learning (ML), statistical data count models maintain their prevalence in analyzing crash data due to their inherent capabilities for causal inference and interpretability [87]. Nonetheless, achieving a balance between improving predictive capabilities and preserving causal inference continues to be a formidable challenge [140].

Advanced heterogeneous models, such as those with random parameters, have emerged as efficient approaches to perform prediction and inference analysis [125]. These probabilistic heterogeneous models can take various forms, from Poisson regression [13], Generalized Poisson (GP), Negative Binomial (NB), and its variants [90]. The NB model is particularly favored for its simplicity and capability to manage overdispersion. However, if the data is under-dispersed, the Conway-Maxwell-Poisson (COM-Poisson) model or the GP model can be a viable alternative [15], [148]. Despite its ability to manage both underdispersion and overdispersion, the COM-Poisson model is often sidelined due to challenges in its implementation. One such challenge is the lack of a closed-form expression for its moments, necessitating high-dimensional Monte Carlo simulations or numerical approximations [35], [45]. Additionally, its practicality is often disputed because of the rarity of under-dispersion occurrence in crash data [153]. On the other hand, non-closed form models that employ Bayesian computation typically focus on addressing model stochasticity to account for an increased number of zero observations in the data [89]. Their time-consuming computations preclude extensive examination of likely contributing factors, and limits extensive exploration and exploration of alternative specifications [3], [14]. Consequently, the process of constructing hierarchical models considering simultaneously multiple important aspects such as dispersion, unobserved heterogeneity, and correlations is intricate. It entails a multitude of modeling decisions which, particularly in the absence of well-founded hypotheses, can introduce bias which, in turn, can impact the precision of parameter estimates [83], [143].

Recent advances in data-driven ML methods, such as random forrests (RFs) [7], [24], [86], support vector machines (SVMs) [31], [102], [147], and neural networks (NNs) [9] have paved the way for removing the burden of significant hypothesis testing and enhancing prediction capabilities. However, their utility is often hampered by their limited ability to reveal causality and provide substantial inferences. These models, often dubbed as ‘black boxes’, present a challenge in identifying which factors influence the dependent variable(s), thus hindering the development and implementation of countermeasures to mitigate crashes [95]. Furthermore, ML methods may struggle with overfitting problems and require meticulous determination of sensitive hyperparameters [84]. To address these concerns, machine learning models have been integrated with metaheuristics to efficiently determine the MLs hyperparameters and prevent overfitting [26], [33], [38], [42]. However, this approach merely transposes the problem into another domain of optimization algorithms, where finding appropriate hyperparameters remains a challenge [71]. Consequently, despite the significant effort they require in model development, statistical models are preferred for road-safety analysis [21].

Nonetheless, developing advanced heterogeneous crash frequency models is a laborious process, demanding the analyst to test numerous hypotheses. These hypotheses encompass various modeling aspects, including but not limited to (I) likely crash contributing factors, (II) variable transformations, (III) functional forms, as well as dealing with unobserved heterogeneity using random effects, random parameters, mixing models, and/or (IV) distributional assumptions. Analysts must construct and iterate through these hypotheses until a satisfactory and useful model is achieved. This process is heavily reliant on knowledge, experience, and an ad hoc approach, which risks subjectivity and bias unless a systematic and comprehensive process is employed [17], [30], [109]. Just as metaheuristics have aided ML models in discovering hyperparameters, they can be harnessed to assist in the analysis by systematically and extensively testing multiple model specifications while preserving interpretability [20]. Adopting a systematic and comprehensive approach with an optimization framework enables testing a larger set of specifications than an analyst could manually, leading to a more informed decision-making process [107], [154]. Without such a systematic and comprehensive approach, errors may be introduced, resulting in misspecifications, estimation biases, and incorrect inferences and predictions [119]. Thus, there is need for an optimization framework capable of addressing the four core aspects of hypothesis testing listed above in a systematic and comprehensive manner.

Hypotheses I and II involve selecting the likely crash contributing factors to include in the model, and the type of associated transformations that can be applied, respectively. They are part of determining a functional form seeking to capture how independent variables influence crash occurrences. As part of the functional form, most literature has used variants of the log-linear expression [13], [54], [115]. However, due to their linear relationship, these forms cannot explain peaks, valleys, or inflection points [94], [138]. Alternative functional forms such as proportional, exponential, inverse and polynomial are used to overcome this problem and other alternatives have been proposed by Hauer [60].

Hypotheses III centers around the means of capturing the functional form, integrating probability assumptions and managing unobserved heterogeneity inherent in the data. Among various statistical methodologies, Poisson regression serves as a foundational approach because crashes often adhere to a Poisson process under homogeneous conditions [91]. However, the Poisson approach may fall short when it comes to dealing with an excess of zero observations in the data [96].

While some research advocates the use of Zero-Inflated (ZI) models to account for high frequency of zero-crashes and the corresponding enhanced statistical fit [37], [93], [144], this approach is not without significant limitations, especially regarding its inability to accurately portray causality underlying the crash generating process. It is essential that statistical methods and their underlying assumptions are applied with discernment to maintain model parsimony and withstand rigorous logical scrutiny [92].

For instance, ZI models often prioritize data fitting at the expense of elucidating the underlying crash occurrence process [90]. That is, they categorize road segments into crash-free or crash-prone sections. However, an excess of zeros does not necessitate adopting a dual-state process unless such a process genuinely exists, which is typically not the case with crash data [120], [139]. Therefore, if causality is a priority, the use of ZI models for crash analysis and interpretation is strongly discouraged [90], [91], [92], [139], [151].

As such, when dealing with datasets primarily composed of zero values, it is essential to employ specialized techniques, such as distributions with properties tailored to accommodate this specific aspect [4]. The Lindley distribution, for instance, is characterized by a long tail that can effectively handle data with few or a large number of zero observations [32]. Behara et al. [21] successfully estimated crash frequency models for heavy vehicles using the Lindley distribution couple with NB models. The motivation for considering the Lindley distribution, as they reported, was the large number of zeros present in their data [21].

Lord and Geedipally [89], Reddy Geedipally et al. [113] were among the first to apply the NB-Lindley approach to crash data. Their research illustrated that NB-Lindley models were more effective for analyzing traffic crash data, particularly when the observations were highly dispersed or contained a large number of zeros. Various mixture variants, such as NB-Generalized Exponential [130], COM-Poisson [2], Poisson-lognormal [108], [120], and Poisson-Weibull [16], among others [155], [156], offer potential alternatives for handling an excess of zeros. However, it is important to weigh the methodological disadvantages associated with these mixture models. As emphasized by Lord and Geedipally [89], the major limitation of these models is the absence of closed-form expressions. This necessitates the use of Bayesian approximation or complex Monte Carlo simulations, methods that are computationally demanding and may limit an extensive analysis.

Acknowledging these challenges, Lord and Mannering [90], Lord et al. [91] suggested that for datasets with a high proportion of zeros, the most theoretically justifiable modeling approaches involve selecting appropriate time/space scales for analysis [7], incorporating an enhanced set of explanatory variables [4], and/or accounting for unobserved heterogeneity effects [65]. Unobserved heterogeneity is a frequent issue in crash data, often arising from inconsistent data collection, measurement errors, underreporting, and unobservable factors [150]. To effectively capture this underlying variability, the use of hierarchical statistical models is recommended [9], [55].

Random-effects models provide a solution for unobserved heterogeneity by accounting for group-level variations in the data [10], [41]. They build upon fixed-effect models by modeling the variability between different groups in the analysis [52], [153]. However, these models focus solely on group-specific differences, operating under the assumption that individual-specific heterogeneity is unrelated to the likely contributing factors. This approach may fall short when handling crash data, where clear grouping characteristics may not always be evident. This limitation makes random-effects models less suitable in practice [76]. In contrast, models with random parameters allow to account for individual-level differences [21], [22]. This approach effectively captures heterogeneity, especially when group distinctions are less clear and unobserved heterogeneity is largely individual-based [111].

While random parameters/effects models provide valuable insights, they do not inherently account for spatial or temporal effects [118], [133]. This is a critical consideration in crash frequency models, as roads in close proximity often share common characteristics, and these spatial and temporal correlations can significantly influence model estimation [85], [112]. Recognizing the importance of these factors, Li et al. [86] proposed a Bayesian model that incorporates spatial and temporal effects. This model outperforms traditional approaches by capturing a more nuanced association between covariates and crash risk. Ignoring spatial and temporal correlations can skew the inferences drawn from models and hinder their predictive ability for future safety improvements [61], [65], [95]. However, it is worth noting that data limitations might prevent modeling of these effects. In such cases, an alternative approach involves extending random parameters to account for correlations between different sources of unobserved heterogeneity [65], [72], [134], [152]. Including a correlation structure among the random parameters requires the addition of more terms than those used in traditional models. If these random correlated random parameters are not significantly correlated with each other, the results could be an overfitted model [117].

Building on this understanding of the limitations and challenges of current methodologies, another alternative to better capture unobserved heterogeneity is to estimate model parameters such as means and variances as a function of observed likely contributing factors [21], [22], [118], [133]. Models with heterogeneity in their means and/or variances provide additional flexibility by capturing underlying unobserved variability through a combination of external contributing factors. For example, certain road conditions may be influenced by other contributory factors, providing more information about the crash generation process. Despite the availability of suitable and extensive data, modeling spatio-temporal heterogeneity in means and correlations can be highly complex and time-consuming [7]. If these complexities are not correctly identified during model development, they can lead to potential issues, including multicollinearity, model misspecification, and intricate result interpretation [64].

The aforementioned methodologies present another layer of complexity. While widely utilized in traffic safety studies, models with random parameters and their extensions carry a significant modeling assumption, namely the need to choose specific distributional properties. These inherent parametric assumptions, particularly those concerning the choice of distribution, embody hypothesis testing IV. If inappropriate distributions are selected, they can impose restrictive constraints, potentially undermining their effectiveness [96]. For instance, when constructing a hierarchical model, random parameters are often estimated using a normal distribution [59]. However, when the properties of the normal distribution, such as unbounded support and symmetry, are violated, it can result in illogical parameter signs. This is particularly noticeable in hierarchical models dealing with sparse data or small group sizes as using normal draws in these situations can result in misspecification and inaccurate results [95]. To mitigate these issues, alternative distributions have been proposed to better capture data characteristics [34]. These include handling skewness and heavy tails, reducing sensitivity to outliers, improving variability estimation through asymmetry incorporation, and better boundary handling [47], [51]. The log-normal distribution is often considered an alternative for controlling coefficient signs [101]. However, its long tail can potentially bias mean values, thus affecting other parameter estimates in the modeling process [121]. Non- and semi-parametric distributions can relax some of these limitations such as the need to pre-specify the shape or functional form [136]. However, these distributions, in the absence of a well-defined functional form, lead to numerous complications. Notably, they can suffer from ill-posedness [63], present computational challenges, and complicate hypothesis testing along with their subsequent validation, and escalate the risk of overfitting [18], [82]. Additionally, the interpretability of results, especially in the context of non-parametric models, becomes more convoluted [114]. While the Lindley distribution has not traditionally fit the distributional assumptions for random parameters, its characteristics, similar to those of a log-normal distribution, could potentially yield sound results [116]. Nonetheless, it is important to carefully consider the trade-offs and suitability of distributions in statistical analyses, as they can significantly impact the soundness and reliability of the results [20].

As a result, analysts, who typically work under time constraints, need to consider multiple hypotheses concurrently. While Bayesian methods are frequently employed for their ability to manage complex models, their simulation time, influenced by sample size and model complexity, can present a significant hurdle [90]. An efficient alternative for heterogeneous models is maximum simulated log-likelihood (MSL) [129]. This method can effectively estimate with closed-form functions that are often readily available [153]. When such functions are accessible, the difference in results between the Bayesian and MSL methods is negligible. The importance of prioritizing computational efficiency is emphasized by utilizing closed-form functions, which offer considerable advantages in the MSL approach. This permits testing a larger number of alternative hypotheses [54]. The choice of MSL over Bayesian estimation ultimately leads to improved results, as demonstrated by the additional proposed hypothesis tested. This advancement paves the way for considering diverse and significant hypotheses, facilitating key modeling decisions for prediction and interpretation [44].

## 2 Research gap and proposed contribution

While data-driven techniques have demonstrated the ability to provide robust statistical predictions with minimal hyperparameter testing, they are inherently constrained by their inability to ascertain causal relationships. Furthermore, these models often produce outputs that are complex and difficult to interpret [49], [50], [124]. Hence, there is a significant gap in the literature for an optimization-centric framework for crash count analysis that enables interpretability while considering simultaneously multiple important modeling aspects [107], [109], including the likely contributing factors, transformations, random parameters along with their distributional assumptions, and the probabilistic model type.

In the specific context of crash frequency, a relevant approach was provided by Veeramisti et al. [132], where cluster-wise safety performance functions (SPFs) were estimated using a Simulated Annealing (SA) algorithm. Their framework uniquely allows for the simultaneous estimation of the optimal number of clusters alongside the corresponding safety performance functions. However, their scope was limited because the SPFs did not consider multiple potential contributing factors beyond exposure, non-linearities, nor random parameters. Similarly, Beeramoole et al. [20] developed an extensive hypothesis testing framework for the estimation of discrete outcome models, wherein an optimization approach discerns explanatory variables while preserving interpretability and testing advanced specifications.

In response to these identified gaps, this paper proposes an optimization framework including a detailed mathematical programming formulation and alternative solution algorithms for the estimation of crash frequency models consistent with the motivation by Beeramoole et al. [20] for the development of discrete outcome models. This study makes significant contributions to the literature in the following ways:

• A mathematical programming formulation that facilitates guided and extensive hypothesis testing for the estimation of data count models. This formulation concurrently considers various modeling aspects, including potential contributing factors, transformations, fixed effects, random parameters along with their distributional assumptions, and the probabilistic model type. The simultaneous consideration of these modeling aspects as decision variables allows for extensive testing that can reveal insights that an analyst might overlook, thereby minimizing error and saving time. This program is designed to minimize overfitting and human bias.
• The introduction of a bi-level optimization framework that incorporates three alternative metaheuristic solution algorithms for generating and testing specifications estimated using maximum simulated log-likelihood.
• Experiments with real data to demonstrate the advantages of the extensive hypothesis testing framework compared to existing published models. The proposed framework facilitates the extraction of similar insights at lower costs while also capturing causality.
• Experiments with synthetic data to validate the robustness of the proposed optimization framework and solution algorithms.

The proposed framework serves as a potent instrument for exploring and estimating specifications across a broad range of applications. It is competent in managing complex scenarios involving numerous potential contributing factors, varying types of data dispersion, and a wide array of potential distributions that are not commonly tested. While models estimated using this framework still require further analysis, the framework simplifies the process of understanding causality and deriving meaningful conclusions.

## 3 Methodology

A non-linear mixed integer mathematical programming formulation for the estimation of data count models is proposed. This formulation involves the testing of multiple modeling aspects, including the probabilistic model type, likely contributing factors and their transformations, as well as random parameters and their corresponding parametric distributions. These aspects are represented as decision variables.

### 3.1 Notation and definition

The notation used to discuss the mathematical programming formulation is presented in Table 1.

**Table 1:** Notation: Extensive hypothesis testing for the estimation of crash data count models.

Data	

*i*	Subscript to denote a road segment observation.
I	Set of all observations, I={1,2,…,i,…}.
K	Set of likely contributing factors, K={1,…,k,…,K}.
M	Set of model types, M={Poisson, NB, GP, …}.
$M^{E}$	Set of model type error assumptions, capturing the distributional properties of the rate parameter
D	Set of distribution types, D={Gamma, Uniform, Normal, Triangular, Lindley, …}.
T	Set of mathematical transformations, T={ln(),sqrt(),exp(),squared(),cubed(),factorial(),arcsinh(),…}.
*x*_*k*_	Vector of observations associated with likely crash contributing factor *k*.
*c*	Upper and lower bound values for variable transformation.

Decision Variables	

*X*	Matrix of transformed data of size K×I
*X*_*i*_	Vector of all transformed variables within observation *i*.
*β*	Vector of coefficients associated with likely contributing factors; *β* = {*β*_1_,…,*β*_*k*_,…,*β*_*K*_}.
***f***	Vector of probability functions for the assumed distributional assumptions with random parameters; ***f*** = {*f*_1_,…,*f*_*k*_,…,*f*_*K*_}, $\forall f_{k}\in D$.
***τ***	Vector of possible transformations to be applied on *x*_*k*_; ***τ*** = {*τ*_1_,…,*τ*_*k*_,…,*τ*_*K*_}, $\forall k\in K,\tau_{k}\in T$.
*b*_*k*_	Mean coefficient for likely contributing factor *k*.
*ω*_*k*_	Standard deviation for likely contributing factor *k*.
*α*_*k*_	Indicator variable taking value 1 if likely contributing factor *x*_*k*_ is included in the crash count model; 0 otherwise.
*r*_*k*_	Indicator variable taking value 1 if likely contributing factor *x*_*k*_ is modelled with random parameters; 0 otherwise.
*κ*	Vector of indicator variables, taking value 1 if the model is from selected set *M*; *κ* = {*κ*_1_,…,*κ*_*m*_,…,*κ*_*M*_}, ∀m∈M.
*ζ*	Number of parameters in the model estimation.
*q*	Indicator variable taking value 1 if model *κ*_*m*_ has dispersion parameter, 0 otherwise.

Pre-specifications set by the Analyst	

$\overset{ˆ}{\alpha_{k}}$	Indicator input, if value takes 1 then likely contributing factor *x*_*k*_ is pre-specified to be included in the model; excluded if 0 otherwise and retracted if undefined.
$\overset{ˆ}{r_{k}}$	Indicator input, if value taking 1 then likely contributing factor *x*_*k*_ is pre-specified to have random parameters; excluded if 0 and retracted if undefined.
$\overset{ˆ}{f_{k}}$	Pre-specified probability function which is used as an assumption for random parameters *r*_*k*_.
$\overset{ˆ}{\tau_{k}}$	Pre-specified data transformation to be used if associated with contributing factor *x*_*k*_.
$\overset{ˆ}{\kappa_{m}}$	Pre-specified probabilistic model.

### 3.2 Problem formulation

The goal of this research is to develop a hierarchical structure in an objective oriented manner, which will facilitate the estimation of crash frequency models. Determining the structure is only part of the process; an approach to calculate the coefficients of the proposed structure is also required. As such, the problem is formulated as a bi-level mathematical optimization program. The objective function of the upper-level aims to minimize the Bayesian Information Criterion (BIC), as denoted in Equation (1). The BIC is widely used in statistics for model selection because it penalizes for adding parameters thereby precluding overfitting while facilitating balance between complexity and accuracy [11], [25], [137]. Moreover, the BIC is frequently utilized across numerous studies owing to its asymptotic consistency and demonstrated efficacy in producing robust results in a variety of research domains [20], [27], [74], [75], [80], [131], [137]. It is even more fitting than alternatives such as Akaike's Information Criterion (AIC), as it better encapsulates model complexity and accuracy [80].

(1) MinBIC=−2ln(L)+ζln(|I|)

where *L* is the likelihood of the model estimation across each observation.

The lower level objective function involves maximizing the MSL by finding all significant estimable parameters in the model. The simulated log-likelihood function is described in Equation (2), wherein $\kappa_{m}$, is an indicator variable. Only one $\kappa_{m}\in M$ can be activated, which helps specify that the probability function adopts the properties of the model type m∈M. For instance, this may involve potentially assuming Poisson, NB, or GP distributions. Further, in Section 6, the probability assumptions of the set of considered models are formally declared and defined. Due to the lower-level dependency, the decision variables primarily concentrate on proposing the structure, while the coefficients are solved in accordance to the lower-level objective denoted in Equation (2).

(2) $$ln(L)=\sum_{i\in I}ln\left[P(Y_{i}=y_{i}|\lambda_{i},m)\right]\;\forall m\in M:\kappa_{m}=1$$

The proposed objective is to parsimoniously assist in predicting crashes based on the characteristics of different road segments, capturing the underlying relationships. To achieve this, a statistical modeling framework is employed based on the assumption of a specific model type, denoted as M. The first step in our approach is to estimate the parameter *β*, which represents the coefficients associated with various contributing factors influencing crash occurrence.

Once the parameter *β* is estimated, the mean number of crashes on each road segment observation can be determined using Equation (3). However, it is important to note that our model acknowledges the presence of model errors, which can arise due to various factors not accounted for in the model. This is addressed by incorporating a flexible representation of the model error term, denoted as $\epsilon_{i}$, in Equation (4). This equation allows to capture and quantify the uncertainties associated with the model predictions.

To further explore the assumptions of our statistical model, the error term is sampled from subsequent rate parameter assumptions in an extended model $M^{E}$, as shown in Equation (4). This allows to evaluate the robustness of our modeling approach and investigate the impact of different assumptions on the estimated crash rates.

While Equation (3) provides the mean estimate of the coefficients at the observation level, we are also interested in examining the coefficients associated with the contributing factors and their standard deviations in more detail. For this purpose, we introduce Equation (5), which represents the formulation of the coefficients $\beta_{k}$. The decision on whether to treat $\beta_{k}$ as fixed or include random parameters is determined through an optimization process. Random parameters can be sampled using various distribution assumptions, such as normal, gamma, Lindley, among others, which are defined in D. This flexibility allows us to conduct an extensive analysis and capture important underlying relationships.

Finally, the covariates *X* in Equation (3) are transformed using functions denoted as $\tau_{k}$ in Equation (6). These transformations are applied to ensure appropriate modeling of the covariates and improve the interpretability of the estimated coefficients.

By employing this approach and leveraging the equations presented below, a concise mathematical description of crash occurrence on different road segments is presented, incorporating uncertainties and exploring the relationships between contributing factors and crash rates.

The mathematical program is subject to the following constraints:

(3) $$\lambda_{i}=\exp(X_{i}\beta +\epsilon_{i})\;\;\forall i\in I$$

(4) $$\epsilon_{i}=m^{\prime }\;\;\forall m\in M,\exists m^{\prime }\in M^{E}:\kappa_{m}=1,\forall i\in I$$

(5) $$\beta_{k}=\alpha_{k}b_{k}+r_{k}f_{k}(\omega_{k})\;\;\;\forall k\in K,f_{k}\in D$$

(6) $$X=\tau_{k}(x_{k})\;\;\forall k\in K,\tau_{k}\in T$$

(7) $$\alpha_{k}=\left\{ \begin{array}{cc} 1, & \text{if }x_{k}\text{ is included;}\\ 0, & \text{otherwise} \end{array}\right.\forall k\in K$$

(8) $$r_{k}=\left\{ \begin{array}{cc} 1, & \text{if }x_{k}\text{ is signified having random parameters;}\\ 0, & \text{otherwise} \end{array}\right.\forall k\in K$$

(9) $$\alpha_{k}\geq r_{k}\forall k\in K$$

(10) $$-c\leq \tau_{k}(x_{k})\leq c\forall k\in K,\tau_{k}\in T$$

(11) $$\zeta =\sum_{k\in K}(\alpha_{k}+r_{k})+q$$

(12) $$\sum_{m\in M}\kappa_{m}=1$$

(13) $$q=\left\{ \begin{array}{cc} 0, & \text{if }\kappa_{1}\text{ = 1 (signifying Poisson);}\\ 1, & \text{otherwise (dispersion parameter present)} \end{array}\right.$$

The following constraints are imposed by the analyst (Pre-specification constraints):

(14) $$\alpha_{k}=\left\{ \begin{array}{cc} 1, & \text{if }\overset{ˆ}{\alpha_{k}}=1\text{ (analyst forces inclusion);}\\ 0, & \text{if }\overset{ˆ}{\alpha_{k}}=0\text{ (analyst forces exclusion);} \end{array}\right.\forall \overset{ˆ}{\alpha_{k}}\in K$$

(15) $$r_{k}=\left\{ \begin{array}{cc} 1, & \text{if }\overset{ˆ}{r_{k}}=1\text{;}\\ 0, & \text{if }\overset{ˆ}{r_{k}}=0\text{;} \end{array}\right.\forall \overset{ˆ}{r_{k}}\in K$$

(16) $$\kappa_{m}=\left\{ \begin{array}{cc} 1, & \text{if }\overset{ˆ}{\kappa_{m}}=1\text{;}\\ 0, & \text{if }\overset{ˆ}{\kappa_{m}}=0\text{;} \end{array}\right.\forall \overset{ˆ}{\kappa_{m}}\in M$$

(17) $$f_{k}=\left\{ \begin{array}{cc} \overset{ˆ}{f_{k}}, & \text{if }\exists \;f_{k}\text{;}\\ f_{k}, & \text{if }∄\;\overset{ˆ}{f_{k}} \end{array}\right.\forall k\in K,f_{k}\in D$$

(18) $$\tau_{k}=\left\{ \begin{array}{cc} \overset{ˆ}{\tau_{k}}, & \text{if }\exists \;\tau_{k}\text{;}\\ \tau_{k}, & \text{if }∄\;\overset{ˆ}{\tau_{k}} \end{array}\right.\forall k\in K,\tau_{k}\in T$$

Equation (7) and Equation (8) are used to include likely contributing factors $x_{k}$ and to denote if random parameters are associated. It is crucial to remember that random parameters can only be estimated if fixed variables are included in the model. Equation (9) ensures that if a likely contributing factor $x_{k}$ is fitted with random parameters, its fixed effect is also incorporated into the model. Equation (6) transforms the likely contributing factors $x_{k}$ into a new form, dependent on the variable $\tau_{k}$, where $\tau_{k}$ can be any transformation in set T. To prevent potential undesired transformations, Equation (10) ensures a transformation can only be applied if all values specific to the likely contributing factors are within the upper and lower boundaries of the specified cost *c*. Additionally, Equation (10) prevents transformations from being applied if errors occur, such as a logarithmic transformation of a negative value. Given the diverse characteristics of likely contributing factors, an analyst might wish to minimize or enforce specific transformations on each of them. In such situations, Equation (18) is utilized to reduce the number of hypotheses tested for data transformations via analyst assistance.

Random parameters depend on the parametric assumption of a distributional form, which is included within set *D*. The variable $f_{k}$ selects the most suitable distribution within set D for element k∈K. Variable $f_{k}$ is used in Equation (5). To streamline the decision-making process in this mathematical program, an analyst can provide guidance. Equation (17) enforces the inclusion or exclusion of specific distributions that strongly correlate with the contributing factor $x_{k}$. Likewise, Equations (14) and (15) allow an analyst to dictate which contributing factors must be included or excluded in the final model along with their random parameters.

Equation (12) dictates the model type used for estimation, such as Poisson, NB, or GP; only one model type can be selected. An analyst may also wish to include or exclude a particular model from consideration; Equation (16) ensures that a probabilistic model is included or excluded, if specified by the analyst. Equation (13) computes the number of additional parameters used to estimate a model. Equation (11) tallies the number of parameters in the model for estimation, which influences the objective function.

## 4 Extensive hypothesis testing algorithm

The objective functions detailed in Equations (1) and (2) are subject to a large array of interdependent decision variables. Consequently, the problem of capturing the crash process involves nonlinear decision-making. The estimation time required for a specific specification of the chosen set of solutions is substantial and scales with the number of crash observations, potential contributing factors, and the number of random parameter sample draws. This calls for an efficient optimization strategy capable of escaping local minima in search of an optimum.

While Xu et al. [147] and Guido et al. [56] demonstrated the efficacy of Genetic Algorithm (GA) and Particle Swarm Optimization (PSO) in solving similar problems, other metaheuristics have been explored. These include Harmony Search (HS), SA, and Differential Evolution (DE) [8], [36], [73], [77]. These methods, which have fewer hyperparameters compared to the GA and PSO employed in Xu et al. [147] and Guido et al. [56], are hypothesized to be less sensitive to hyperparameter tuning due to the smaller number of individual hyperparameters requiring adjustment. All developed algorithms were found to be adequate for conducting extensive hypothesis testing and efficiently navigating the search space. These metaheuristics do not require derivative information, have the capability to escape local optima while intensifying search to ensure convergence, and require only a few easy-to-handle hyperparameters [103]. Despite their relative ease of tuning, covering arrays were employed to probe their sensitivity and ensure proper adjustment [68].

Discrete versions of the algorithms DE and HS are implemented, along with SA. The search process for each metaheuristic does not guarantee finding an optimal solution, as exploring the entire solution space within a practical timeframe is unrealistic due to non-linearities and a large number of interdependent decision variables [104]. However, previous studies have demonstrated that metaheuristics are problem-independent and effectively solve various optimization problems within practical time frames, either optimally or very close to optimality [28], [29], [79]. Hence, the selection of a metaheuristic search-based approach as the solution algorithm is justified due to its flexibility in generating complex heterogeneous models, thereby facilitating efficient hypothesis testing [20]. Metaheuristics can accommodate complex hierarchical structures, making them well-suited for handling challenging scenarios such as selecting likely contributing factors, random parameters and their distributional characteristics, transformations, which exhibit significant non-convexity that cannot be addressed by traditional mathematical solvers [40] such as CLPEX [66] or Gurobi [57].

All metaheuristic algorithms developed in this study operate within a discrete framework. SA is inherently discrete, while the discrete versions of DE and HS take inspiration from the works of Wu et al. [142] and Alia and Mandava [8], respectively. To ensure a fair comparison, all algorithms have been adapted to incorporate a time-based stopping criterion.

#### 4.0.1 Harmony search

HS takes its inspiration from the improvisation process of jazz musicians, where initial harmonies are refined over time through pitch adjustments to achieve an ideal harmony [48]. HS has three main hyperparameters, which include harmony memory size (HMS), pitch-adjustment rate (PAR), and harmony memory consideration rate (HMCR). A discrete variant of HS, influenced by Kattan et al. [73], was employed for this study, due to our decisions being all discrete.

The distinguishing feature of this variant from the classical HS algorithm is the adoption of an index-based implementation called the pitch-adjustment index (PAI), which replaces the continuous hyperparameter known as the pitch-adjustment proportion (PAP). The PAI can be represented by a discrete variable that controls the maximum allowable movement of indexes to the current position when employed in an ordered set of decisions. Because of this for each index of our chromosome, were a decision is required, there is a further indexed selection to determine the decision. To perform this operation, the function $\ell_{p,j}$ is utilized, which returns the index of the decision currently imposed by solution *p* at position *j*. This index corresponds to a decision that is part of the sequence of ordered decisions. Formally, if $L_{j}=\{l_{1},l_{2},\ldots ,l_{n}\}$ is the set of all possible decisions at element j, ordered by some criterion, and $S_{p}=\{s_{p1},s_{p2},\ldots ,s_{pm}\}$ is the sequence of decisions for solution *p*, then $\ell_{p,j}$ maps the *j*-th element of $S_{p}$ to its corresponding index in $L_{j}$. That is, if $s_{pj}=l_{k}$, then $\ell_{p,j}=k$, ensuring that $s_{pj}\subseteq \{d_{1},d_{2},\ldots ,d_{k}\}$.

At the start of the algorithm, the initial harmony memory set, denoted as harmony memory (HM), is randomly initialized for all independent decisions or preconfigured according to analyst preferences. The size of HM is determined by the hyperparameter HMS. This set represents a collection of model specifications, where each specification represents a potential solution to the mathematical program that requires optimization.

**Figure 1:**
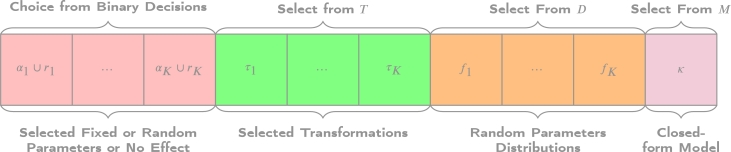
A chromosome representation for the decision variables. The length is predominantly dependent on the number of potential contributing factors (K) that are available.

To evaluate the quality of each solution, the BIC is utilized as the objective function. However, other goodness-of-fit measures could easily be substituted in its place. The decision variables consist of the selection of likely contributing factors, transformations, randomly sampled heterogeneously declared contributing factors from a choice of different distributions, as well as the closed-form model that encompasses the overall structure. These decision variables need to be determined. Modifying these decision variables will result in changes to the BIC, as they drive the modeling equations to estimate the model, directly influencing the BIC and shaping the search space. Thus, each independent decision can take distinct values from their respective sets. For instance, the first contributing factor can be transformed using any transformation in the set T, as long as it adheres to the imposed constraints, specifically exemplified in Equation (6). To facilitate the determination of these decisions and enable modifications through metaheuristic operators, a chromosome solution representation is employed. The chromosome structure is illustrated in Fig. 1.

Once the HM is generated, it is sorted based on the objective functions scores of its solutions. Throughout the search process, poorer solutions are discarded in favor of better ones.

The algorithm continues iterating until a maximum time limit, specified by the input MaxTime, is reached. It then returns the best solution found during the exploration. In each iteration, new solutions are constructed by iteratively considering each element, H[j], of the solution vector *H*. The decision-making process is guided by the hyperparameters HMCR and PAR. If a randomly generated continuous value, randomly sampled from U(0,1), is less than or equal to HMCR, a randomly selected element *j* from a solution in the harmony memory (HM) is chosen to update H[j]. Otherwise, a randomized decision id generated for H[j].

**Figure 2:**
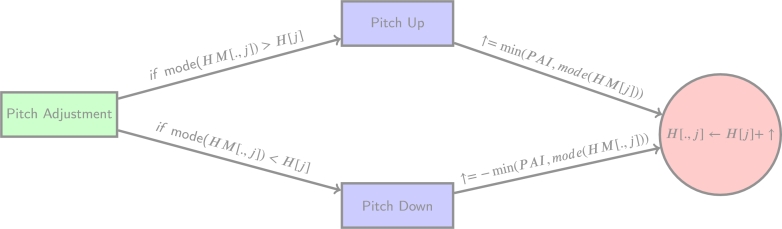
The current solution is shifted towards the direction of the mode of the harmony memory set. It will be shifted by the difference of the current pitch towards the mode at a rate of the minimum discrete number between the difference and PAI.

After updating all elements of *H*, a pitch adjustment may be applied based on the probability specified by the hyperparameter PAR. If a randomly generated continuous value, randomly sampled from U(0,1), is greater than or equal to PAR, a pitch adjustment is performed. This adjustment involves shifting the decision variables according to the direction of the most common pitch adjustment. The PAI determines the rate and range of adjustment for each decision variable. PAI is a discrete index that represents the relative position of a decision variable in the harmony memory. A higher value of PAI indicates a larger range of adjustment from the element of the chromosome that corresponds to the current decision. This adjustment will then shift the decision, allowing for a greater degree of fine-tuning. An example of the pitch adjustment process is provided in accordance with Fig. 2.

Finally, this algorithm was developed for its ability to converge, as only the best solutions within the harmony are retained. This is achieved by evaluating the fitness of the current solution using the function objective(H), and comparing it to the fitness of the worst solution in the harmony memory (HM) obtained through the function objective(HM[HMS]). If the current solution's fitness is less than the fitness of the worst solution in the harmony memory, the current solution replaces the worst solution in the harmony memory. Subsequently, HM is sorted again, ensuring that the worst solution is positioned at the end of the harmony memory.

The overall process of constructing new solutions, evaluating the objective function, and updating HM, and returning the final model for evaluation is depicted in Algorithm 1.

**Algorithm 1:**
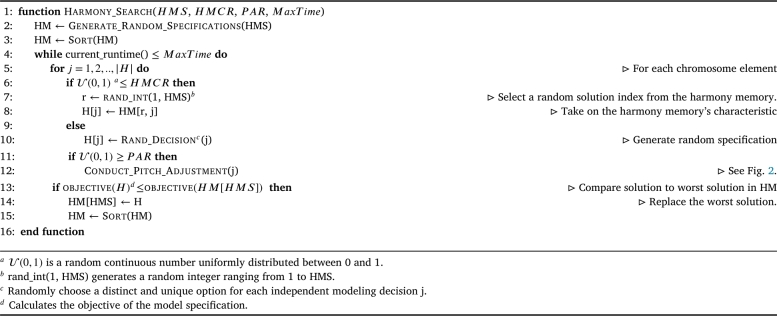
Harmony Search.

#### 4.0.2 Simulated annealing

In the implementation of Simulated Annealing SA, several hyperparameters are considered to control the search process. These hyperparameters include the initial temperature ($T_{0}$), cooling factor ($T_{\alpha }$), temperature steps ($T_{s}$), and the stopping criterion (MaxTime).

The initial temperature ($T_{0}$) is a hyperparameter that influences the acceptance probability of transitioning into worse solutions. It is typically set to a large value that exceeds the expected range of the objective function values. A higher initial temperature allows for more exploration of the solution space, while a lower initial temperature focuses on exploitation of local optima. However, due to the unknown and data-dependent nature of the problem, it is challenging to determine a good proxy for the value for $T_{0}$ through manual tuning. To address this, it is recommended to calculate the initial temperature, which helps mitigate the uncertainty surrounding its selection [70]. For this reason, the developed simulated annealing algorithm incorporates a temperature calculation heuristic. The calculation of the initial temperature utilizes the hyperparameter χ=0.5, which has shown success in other optimization problems [5], [19]. This specific choice of *χ* implies that, at the start of the algorithm, approximately half of the solutions are likely to be accepted. This immediate state of equal acceptance establishes a balanced exploration-exploitation trade-off. This approach allows for sufficient exploration of the solution space while also exploiting promising regions. This method of calculating the initial temperature offers several advantages over relying on predefined guidelines. It reduces the sensitivity of the algorithm's performance to the selected value of $T_{0}$. Consequently, it alleviates the burden of manual fine-tuning of the temperature related hyperparameter that need to be determined. The calculation of the initial temperature is demonstrated by Equation (19).

(19) $$T_{0}=\frac{\overset{¯}{\Delta E}}{log(\chi )}$$

The cooling factor in simulated annealing determines the rate of temperature reduction during the algorithm. A smaller cooling factor leads to slower cooling and more exploration, while a larger cooling factor promotes faster convergence but limits exploration. The choice of the cooling factor influences the balance between exploration and exploitation, impacting the algorithm's ability to find global or local optima.

Temperature steps determine the number of iterations or steps taken at each temperature level before decreasing the temperature further. They play a crucial role in defining the length of each annealing stage. Larger temperature steps enable more exploration at each temperature level, allowing the algorithm to search extensively. On the other hand, smaller steps focus on refining and fine-tuning the solutions around the current temperature, prioritizing local optimization.

The maximum time limit, defined as the stopping criterion, directly affects the overall runtime of the SA algorithm. A longer maximum time allows for a more thorough search, increasing the chances of finding better solutions. However, a shorter maximum time may lead to premature termination, limiting the search process and potentially resulting in suboptimal results.

It is important to note that finding the optimal values for these hyperparameters is problem-specific and may require experimentation and tuning. The calculation of the initial temperature is one approach to determine an appropriate starting point for the temperature parameter based on the objective value variance of a randomly selected population of initial solutions. This approach aims to reduce sensitivity to the initial temperature setting.

While the mentioned hyperparameters are important, another crucial component to consider in this metaheuristic implementation is defining the neighboring solutions. In this context, neighboring solutions are generated by applying mutations to the current solution.

To generate a neighboring solution, the algorithm selects a random chromosome element and applies a modification to represent a different decision. This process encompasses various possibilities, such as removing a contributing factor from the model, adding a factor to the model, or changing the distribution of a random parameter.

**Figure 3:**
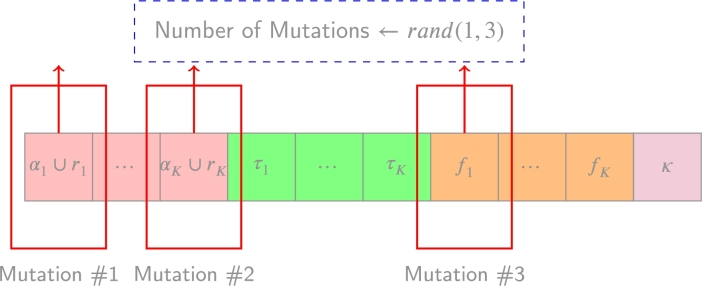
Chromosome, with 3 mutations randomly selected to change its current imposed decision.

In the specific implementation, the number of mutations made in each iteration has been set to be U(1,3). Therefore, at least one, but up to three distinct changes are inherently made to the current solution to form the neighboring solution. These changes contribute to diversifying the search space and facilitate exploration of different configurations. An example of mutation has been made in Fig. 3.

By introducing these random modifications, the algorithm explores the solution space by iteratively moving from one solution to its neighboring solution. This enables the algorithm to search for alternative configurations and potentially discover better solutions that may have been overlooked initially. To further outlines the SA algorithm, this has been illustrated in Algorithm 2.

**Algorithm 2:**
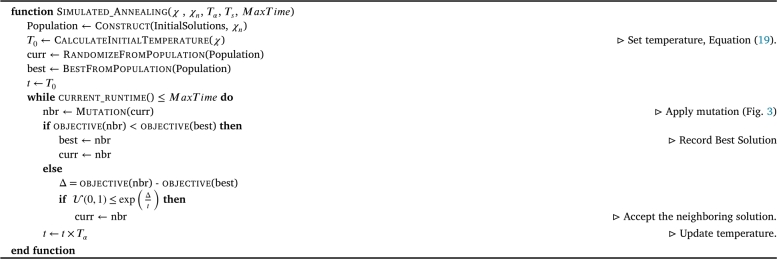
Simulated Annealing.

#### 4.0.3 Differential evolution

Discrete DE bears similarity to HS, but with fewer hyperparameters. The crossover rate (CR) and the population size (PS) are identified as the primary hyperparameters in shaping the creation of a new specification that integrates details from three preexisting solutions.

In discrete DE, the parameter *PAI* is in charge of controlling the permissible movement of the current index for the current decision, behaving identically to the hyperparameter PAI in discrete HS. In the process of updating the solution vector $v_{p,j}$, this applies to all members of the population *p* and for each decision *j*. In this context, $v_{p,j}$ represents the chosen decision for solution *p* within the population pertaining to element *j*. Conversely, $\ell_{p,j}$ acts as a function to identify the current indexed element for solution *p* at indexed element *j*. It symbolizes the current indexed elements of the trial solutions within an ordered set of possible decisions *j* and ensures that various solutions from the population can be altered relative to each other by integrating an indexed decision space.

The equation for $v_{p,j}$ employs the modulo argument to ensure the creation of novel solutions within the confines of the available decision space, without surpassing its boundaries. This equation is elucidated in Equation (20).^1^

Within this function, the parameter *PAI* is explicitly deployed, which can further amplify the space at which decisions can be altered. The equation for $v_{p,j}$ is visually represented in Equation (20)

(20) $$v_{p,j}\leftarrow x_{p,j}[(\ell_{p,j}+PAI(\ell_{p^{\prime },j}-\ell_{p^{\prime \prime },j}))\;\mathrm{mod}\;(|v_{p,j}|)]$$

Here, *p* denotes the index of the currently observed solution within the population, while $p^{\prime }$ and $p^{\prime \prime }$ represent distinct indexes to solutions, each randomly selected from the same population.

The DE algorithm has been illustrated in Algorithm 3.

**Algorithm 3:**
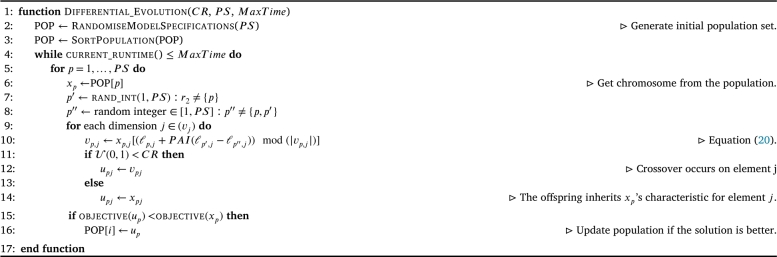
Differential Evolution.

#### 4.0.4 Repairing a specification

To generate model specifications and perform hypothesis tests, one of the developed metaheuristic algorithms could be employed to govern the overarching process. However, the nature-inspired metaheuristic chosen for the solution generation process may introduce proposed specifications that are biased due to the significance levels of individual modeling effects. These biased specifications often include modeling effects that should be rejected. An analyst can typically detect this bias using an ad-hoc process. To address this limitation, a repair process is applied after the estimation of the simulated maximum likelihood and the determination of all the effects significance levels. The repair process is designed to mimic a typical ad-hoc method that an analyst would employ.

In this approach, a single specification is analyzed, reduced, and re-estimated using maximum likelihood estimation. This iterative process continues until stability is achieved. Only then does the metaheuristic utilize that specification, analyze its total fitness, and proceed to the next iteration of the metaheuristic. This incorporation of the repair algorithm ensures that any bias introduced by the previous steps is corrected, resulting in more reliable model specifications.

During this procedure, multiple hypotheses are simultaneously tested, and individual modeling effects may fail to meet the predetermined level of significance. If the associated hypothesis is not rejected, the entire specification is reduced. Components that fail the hypothesis tests are removed, and a new specification is generated and tested. This iterative process continues until convergence is achieved or a predefined number of iterations is reached. The following steps summarize this procedure:

1. Find the maximum insignificant variables and drop them from the chromosome.
2. Re-estimate the model chromosome.
3. Repeat steps 1 and 2 until all variables are significant.
4. Embed the finalized repaired chromosome into the metaheuristics algorithm.^2^

## 5 Implementation

**Figure 4:**
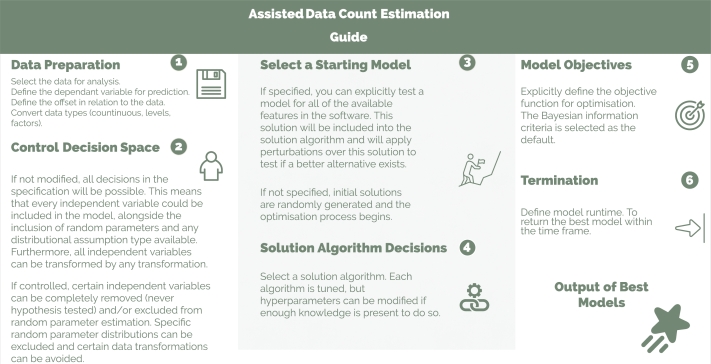
Analyst guide for using the software that implements the proposed framework.

Software has been developed to facilitate the proposed extensive hypothesis testing, aiding in the efficient discovery of likely contributing factors. This is visually represented in Fig. 4, where the hypothesis testing is deployed for estimating data count models. The software, implemented in Python, is available as an installable package [6].^3^ All Computational experiments were conducted using this software, with each experiment assigned to a single thread on an SGI Altix XE Cluster. The software is crafted to test multiple hypotheses that an analyst might unknowingly neglect or be unable to test within the allotted time for modeling. However, the analyst's role remains crucial in providing relevant data, defining the exposure effect, generating an initial solution, and selecting the dependent variable. Furthermore, the analyst needs to identify all potential contributory factors for consideration in the final model. The implementation also allows for a extensive hypothesis testing without using an initial solution provided by the analyst. This represents a case when the analyst is unable to provide any insights regarding likely model structure and relevant characteristics. However, these insights, knowledge and experience are required to assess the quality of the model(s), and analyze and interpret results.

Once the initial data preparation is complete, the implementation guides the analyst in testing various modeling decisions and reducing the search space. For instance, constraints in the mathematical program can exclude certain contributing factors from model generation, effectively narrowing the search space. Moreover, the analyst can predefine which contributory factors must or must not be associated with random parameters. Due to the parametric assumptions required by random parameters, the analyst can choose to exclude certain distributions from the extensive hypothesis testing framework or mandate a specific distribution for a contributory factor associated with random parameters. This flexibility enables the analyst to enforce testing of specific distributions for certain coefficients. For instance, the Uniform distribution could be exclusively tested when estimating the coefficient for the scale random parameters associated with the contributing factor “number of lanes”.

The analyst has the option to supply a starting model (initial solution), which may be modified by the extensive hypothesis testing algorithm. This option allows to consider existing knowledge and traditional modeling outputs while exploring alternative specifications that might have been overlooked. If no starting model is provided, the hypothesis testing algorithm will generate initial specifications randomly and apply perturbations in an efficient manner.

As solution algorithms are somewhat sensitive to their hyperparameters, the analyst has the option to select a specific solution algorithm and its corresponding hyperparameters. Alternatively, the implementation uses default parameters set using results from this study.

The analyst also has the choice to select a preferred objective function. While BIC is implemented by default, alternatives such as AIC and log-likelihood can also be chosen. Lastly, the implementation is designed to allow the analyst to set a maximum runtime (MaxTime) before termination, ensuring that the best solution is reported within the allocated time. The software implementation and its features are summarized in Fig. 4.

## 6 Numerical experiments

The efficacy of the proposed framework and solution algorithms was appraised through numerical tests using three different datasets. These tests aimed to illustrate various facets of the extensive hypothesis testing framework, including but not limited to:

• Evaluating the effectiveness of the proposed approach and objective function using synthetic and actual data;
• Identifying specifications that can accommodate dispersed data;
• Garnering insights and understanding into likely contributing factors for crash estimation;
• Testing multiple hypotheses simultaneously encompassing likely contributing factors, random parameters and their related distributions, data transformations, and the type of probabilistic model; and
• Benchmark results using publish models and open-source data.

For the simulation of the maximum likelihood estimate when modelling random parameters, 200 Halton draws ($H_{\text{draws}}=200$) were employed [58]. The preference for Halton draws stems from their superiority over pure random draws, as demonstrated in various studies [23], [126], [127], [128]. As a result, in the case of a simulated maximum likelihood estimate, Equation (2) is transformed into Equation (21). Given that Equation (21) is a conditional function, it becomes essential to consider appropriately defined modelling decisions in this context. For this experiment, and all others presented in this paper, the set of possible decisions was defined as follows:

• M={Poisson, NB, GP}
• D={Gamma, Uniform, Normal, Triangular, Lindley}
• $M^{E}=\{\text{0,}\;\Gamma \text{(1,}\text{φ}\text{)}\}$
• T={ln(),sqrt(),exp(),squared(),cubed(),factorial(),arcsinh()}

Set M is defined with Poisson,^4^ NB^5^ or GP.^6^ Subsequently, each models inherent error term varies based on the distribution used, making it crucial to account for these errors appropriately with $M^{E}$. This term aids in capturing the model error for each mean equation. Given a rate parameter, denoted as *φ*, is assumed to adhere to model *m* within set M, the models can be generalized as follows:

$$\text{Given }m\Rightarrow \text{Poisson},\text{ then}\;Pr(Y_{i}=y_{i}|\lambda_{i},\text{Poisson})=\frac{\exp\left(-\lambda_{i}\right)\lambda_{i}^{y_{i}}}{y_{i}!}\;\text{Given }m\Rightarrow \text{NB},\text{ then}\;Pr(Y_{i}=y_{i}|\lambda_{i},\text{NB})={\left(\frac{1/\varphi }{1/\varphi +\lambda_{i}}\right)}^{1/\varphi }\left(\frac{\Gamma (1/\varphi +y_{i})}{\Gamma (1/\varphi )+y_{i}!}\right){\left(\frac{\lambda_{i}}{1/\varphi +\lambda_{i}}\right)}^{y_{i}}\;\text{Given }m\Rightarrow \text{GP},\text{ then}\;Pr(Y_{i}=y_{i}|\lambda_{i},\text{GP})=\frac{\lambda_{i}}{1+\varphi \lambda_{i}}{\left(\frac{1+\varphi y_{i}}{y_{i}!}\right)}^{y_{i}-1}\exp\left(\frac{-\lambda_{i}(1+\varphi y_{i})}{1+\varphi \lambda_{i}}\right)$$

(21) $$ln(L)=\sum_{i\in I}ln\left[\frac{1}{H_{\text{draws}}}\sum_{h=1}^{H}P(Y_{i}=y_{i}|\lambda_{i},m)\right]\;\exists m\in M:\kappa_{m}=1$$

### 6.1 Data

The effectiveness of the proposed approach and objective function was evaluated using Monte Carlo simulation, following the work of Geedipally and Lord [46], Hilbe [62]. The simulation enabled a known relationship for model prediction. It also provided opportunity to assess the ability of the proposed framework to accurately identify and reproduce a synthetic model which represents an ideal scenario when the target estimates are known a priori [47]. The synthetic model was created including a constant term, three fixed effects, two random parameters distributed as Normal, and counts following the Poisson process without any transformations. To provide a realistic and fair scenario, five non-significant variables were deliberately introduced into the synthetic data. This step aimed to test whether the extensive hypothesis testing framework could effectively discern the extraneous information as non-relevant. In total, the final synthetic dataset for consideration comprised 2,000 observations, enabling performance evaluation and validation of the proposed approach to identify correctly relevant and non-relevant factors.

The simulation design was implemented through the following steps:

1. Include three fixed and two random parameters as independent variables, complemented by five other non-significant variables intended to introduce random noise. Ensuring that the five key contributing factors are integrated into the model, which will then influence the count response.
2. Simulate the ‘true’ crash occurrences utilizing the coefficients and explanatory variables as outlined in Equation (3). This method is designed to identify three fixed and two random parameter effects. To achieve this, we predefine coefficients on these variables to encapsulate their mixed mean effects, along with the standard deviations for the two random parameter effects. These coefficients are set in advance during the simulation process.
3. Repeat steps 1 and 2, 100 times, a quantity considered sufficient for reliable estimates, as suggested by Lord [88], Roque and Cardoso [115] to achieve credible Poisson count predictions when averaged. Compute the average counts from these 100 repeated trials and employ them as the ‘true’ counts for the experimental dataset.

In addition, two pre-existing datasets along with their published models were employed for testing and benchmarking purposes. Data from Washington et al. [140], originating from 1990 and consisting of road crash records spanning five years, was used. As the data was already collated over these five years, panel data analysis could not be conducted; the number of crashes, with 30 potential contributing factors over 275 roadway segments in Washington State, serves as the predictor. The objective of this experiment was to investigate if the proposed framework could identify a specification that is at least intuitively equivalent to the one in literature. The complexity of this task could potentially have been alleviated using pre-specification constraints, likely to be incorporated by an analyst using their expert knowledge. However, by not using those constraints, an unbiased and more demanding exploration of the search space is assured. Furthermore, the ability of the proposed framework to capture random parameters was illustrated, including potential distributions and other elements considered by the framework but potentially overlooked by an analyst, such as the unique modeling of the distributional assumptions imposed by the random parameters model.

The data, supplied by the Queensland Department of Transport and Main Roads (TMR), was analyzed in Behara et al. [21] and utilized in this study to examine whether analyst pre-specification can enhance the search process within the extensive hypothesis framework. The knowledge, time, and experience demanded by the work in Behara et al. [21] is significant, yet it is postulated that numerous other models potentially exist that could yield more information and do so in a statistically sound manner. Therefore, in this experiment, the specification from Behara et al. [21] is included as an initial solution, with the intent to determine if the extensive hypothesis framework can generate a more sound and better fitted model. The practical aspects of this experiment aim to demonstrate how the combination of analyst effort and the proposed framework can unveil more information. The data comprises 1,478 unique roadway segments in the state of Queensland along with 67 potentially associated contributing factors that may explain head-on collisions.^7^

### 6.2 Covering arrays

The solution algorithm, driven by either HS, SA, or DE, requires tuning of their hyperparameters in each case. Therefore, appropriate hyperparameters must be selected for each algorithm. To ensure a fair comparison between each algorithm and its subsequent hyperparameters, a time limit of 10,000 seconds was imposed, using only one CPU. To test and validate the objective function and determine the appropriate algorithm for the final experiment, the synthetic dataset, which was detailed in Section 6.1, will be utilized.

The hyperparameters and algorithm selected for the final real-data test will be based on an exploration using covering arrays, which are often used to find the most appropriate combination of hyperparameters for successful optimization. Therefore, through this initial experiment, where three algorithms were tested, the appropriate hyperparameters for testing using the final real and synthetic data were discovered.

The guidelines outlined in Simon [122] were followed to determine the initial hyperparameter covering arrays. These covering arrays are outlined in Table 2. For a reminder of the definitions to the hyperparameters, please refer to Table 7.

**Table 2:** Hyperparameters for each algorithm. Tested with five seeded runs.

Algorithm	Hyperparameters covering array		
Harmony Search	*HMS*	HMCR	*PAR*
	{10, 20, 30, 40}	{0.5, 0.6. 0.7, 0.8, 0.9}	{0.3, 0.5, 0.7}
Simulated Annealing	$T_{s}$	$T_{\alpha }$	
	{1, 2, 5, 10}	{0.8,0.85,0.9,0.95,0.975,0.99}	
Differential Evolution	PopSize	*CR*	
	{10, 20, 30, 40}	{0.2, 0.4, 0.6, 0.8, 1.00}	

**Figure 5:**
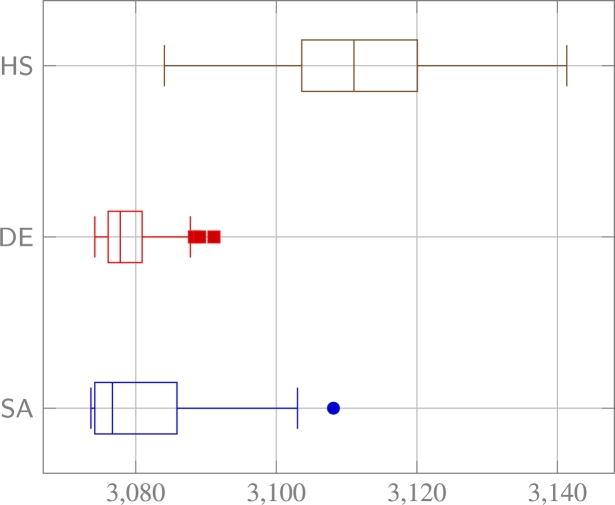
Synthetic data experiment: boxplots of each unique run of an algorithm's hyperparameters, measuring BIC.

The effectiveness of the proposed framework was showcased by utilizing software that implements the proposed extensive hypothesis testing, while acting as an inexperienced analyst with limited knowledge of data count models. As a result, for all tested datasets, only step 1 was performed in Fig. 4. This step involves data preparation without controlling any other decisions. Consequently, the solution algorithm was able to select all potential contributing factors for testing, along with their random parameters and their distributional assumptions, and all currently implemented data transformations, without any pre-specifications to reduce the complexity of the search. The initial results, which were obtained using covering arrays, are presented in Fig. 5. These results clearly show a difference in the quality of the solution achieved by each algorithm. The HS algorithm performed poorly compared to both DE and SA. The SA algorithm achieved the best reported results, but some of its performance was dependent on the selection of hyperparameters. Upon analyzing the data, outliers were observed when the values of $T_{\alpha }=0.8$ and $T_{s}=1$ were used as hyperparameters. This indicates that these hyperparameters did not provide adequate search of the solution space. While some outliers were present in the SA algorithm, excluding them could lead to better results. It was also found that the hyperparameters were not sensitive when the lower-extremes of the results using covering arrays were not considered in Table 2.

Based on the analysis, it is proposed that the problem can be effectively solved when testing other datasets of similar size using a SA algorithm with appropriate hyperparameters. From observing these experiments, it is suggested that the hyperparameters $T_{\alpha }=0.975$ and $T_{s}=5$ be used for the remaining experiments using the SA algorithm.

### 6.3 Convergence analysis

The convergence of final experiments, later subjected to analysis within this paper and representative of the datasets, is illustrated in Figure 6a, Figure 6c. In Fig. 6a, the solution achieves convergence relatively swiftly, with neither the optimal nor incumbent solution demonstrating improvement beyond the 900^th^ iteration. This outcome suggests the effectiveness of the algorithm considering inferior solutions, and subsequently stabilizing within a local-optimal region where superior solutions are absent. Within Fig. 6b, the incumbent solution accepts a multitude of inferior solutions before it converges towards the most efficient known solution. Lastly, Fig. 6c exhibits rapid convergence of the solution to an efficient outcome. However, the algorithm discovers a superior solution at the 680^th^ iteration through exploration, a significant accomplishment considering a probable initial confinement in a local-optima region prior to unveiling an improved solution towards the conclusion. In summary, these results imply the proficient performance of the SA algorithm, when paired with the proposed hyperparameters, in effectively addressing the problem.

**Figure 6a:**
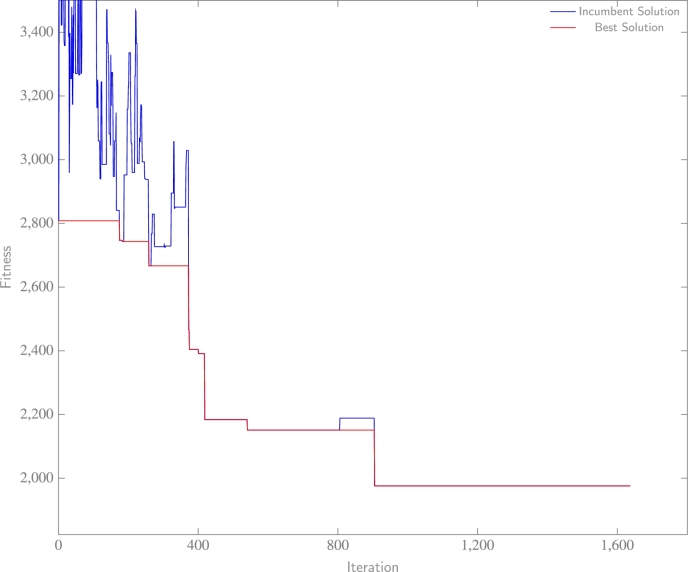
Washington data experiment: fitness (BIC) with respect to each iteration of SA.

**Figure 6b:**
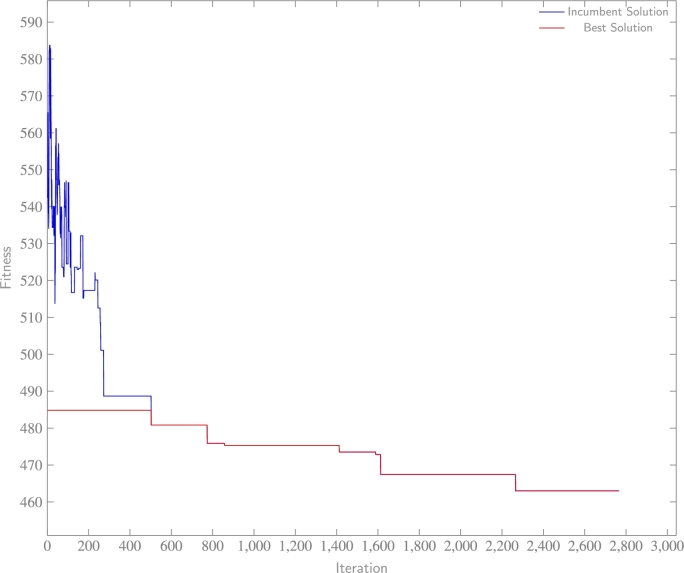
Heavy vehicle experiment: fitness (BIC) with respect to each iteration of SA.

**Figure 6c:**
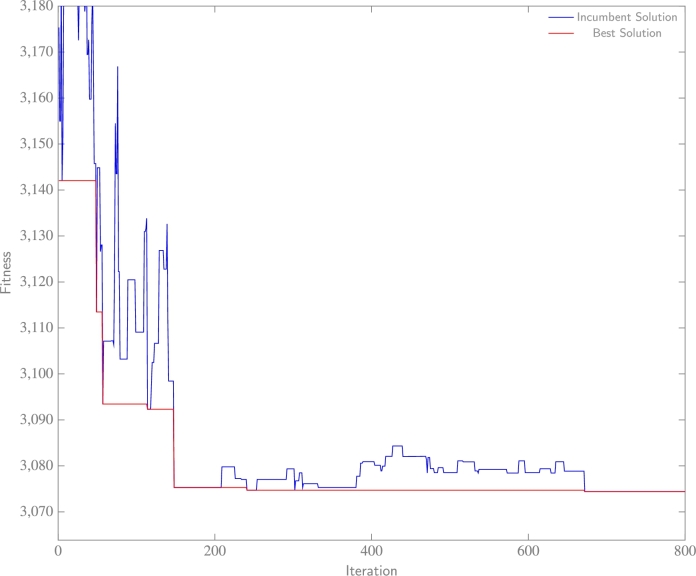
Synthetic data experiment: fitness (BIC) with respect to each iteration of SA.

### 6.4 Synthetic data

The primary objective of this experiment was to evaluate the ability of the extensive hypothesis testing framework to accurately recapture a synthetic model specification. The synthetic model was evaluated using Monte Carlo simulation, as elaborated in Section 6.1. The calculated BIC for the synthetic model was 3075. The model proposed, which was derived through the extensive hypothesis testing framework, was devoid of any pre-specifications and assistance. The proposed framework was able to closely recapture the synthetic specification. As illustrated in this experiment, adopting an optimization-based approach can lead to solutions that closely mirror the underlying crash process. The resultant solution is depicted in Table 3.

**Table 3:** Estimated specification by the proposed extensive hypothesis testing using SA.

Effect	*τ*	Coeff	Std. Err	z-values	Prob |*z*| > *Z*
CONSTANT	no	2.19	0.61	3.57	0.00***
FIXED1	no	-1.02	0.25	-4.12	0.00***
FIXED2	no	2.65	0.55	4.78	0.00***
FIXED3	no	-2.66	0.54	-4.90	0.00***
RANDOM1	no	-2.87	0.60	-4.81	0.00***
RANDOM2	no	-1.25	0.61	-2.04	0.04**

*Scale Parameters for Distributions of Random Parameters*					

RANDOM1 (Std. Dev.) triangular		4.01	0.56	-7.14	0.00***
RANDOM2 (Std. Dev.) normal		0.93	0.23	-4.01	0.00***
Model Type	Poisson				
Log-Likelihood	-1522.38				
BIC	3076.75				

(Standard errors in parentheses).

${}^{⁎⁎⁎}\;p<0.01$; ${}^{⁎⁎}\;p<0.05$; ${}^{⁎}\;p<0.1$.

The proposed model successfully recaptured almost all aspects of the synthetically generated specification, apart from a single contributing factor modeled with random parameters, which was suggested to follow a triangular distribution instead of the normal. This limitation of the extensive hypothesis testing framework is acknowledged, given that not all possible solutions can be tested. Nevertheless, the final model closely mirrored the synthetic specification. Thus, this experiment exemplified that the extensive hypothesis testing framework can identify efficient models without necessitating analyst intervention.

### 6.5 Washington state data

The model identified by the proposed extensive hypothesis testing framework was compared with the model estimated by Washington et al. [140]. Both the benchmark and the model estimated with the aid of SA are included in Table 4. A dictionary containing all relevant effects is provided in Appendix A.3. As in the synthetic experiment, the extensive hypothesis framework was allowed to conduct a comprehensive search of alternative specifications from the data. This approach was chosen to demonstrate whether the proposed framework, in the absence of any prior knowledge, could outperform or estimate comparable models developed by an analyst. A superior specification in terms of BIC was identified by the model proposed through a extensive hypothesis testing framework. This model provided additional insights, including the identification of three likely contributing factors. Moreover, the suggested model highlights the necessity of employing random parameters, as inferred from Washington et al. [140], and deviates through the use of a mixture of distributions (uniform and Lindley). This is an encouraging result facilitated by the proposed extensive hypothesis testing framework, as these distributions may represent better alternatives than the standard normal distribution. For example, the preconceived implications of these distributions on these variables may be limiting the exploration of superior models, while it remains a time-intensive task for an analyst to test all [20], [109]. When multiple distributions were examined, a better fit was identified through the characteristic features of these distributions. Particularly, the Lindley distribution, with its long tail, was noted - a feature not commonly included in the literature for random parameters estimation. This supports that, in the case of average precipitation of rainfall (AVEPRE), the likelihood of significant crashes can be increased across some segments. Even though the fit was enhanced, the final model still requires further analysis to fully utilize the added characteristics. This flexible modeling approach can indeed identify more efficient solutions with a better goodness-of-fit compared to traditional methods. However, it cannot replace the role of an analyst, as the interpretation and analysis of results is still a crucial aspect.

**Table 4:** Specification found by Washington et al. [140, Example 16.3] compared against the proposed framework.

Benchmark			Estimated specification by the proposed extensive hypothesis testing using SA			
Effect	Coeff	Pr |*z*| > *Z*	Effect	*τ*	Coeff	Pr |*z*| > *Z*
CONSTANT	2.81	0.00***	CONSTANT	no	7.66	0.04**
LOWPRE	-0.41	0.00***	ROUTE	sqrt	-0.04	0.00***
GBRPM	-0.05	0.02**	LENGTH	log	0.37	0.00***
FRICTION	-0.01	0.05**	INCLANES	log	-1.14	0.00***
EXPOSE	2.7	0.00***	MXMEDSH	arcsinh	0.73	0.00***
INTPM	0.15	0.01**	SPEED	sqrt	0.66	0.00***
CPM	-0.16	0.00***	AADT	log	0.14	0.01**
HISNOW	-0.18	0.01**	PEAKHR	no	0.03	0.05**
			GRADEBR	no	0.03	0.01**
			MXGRDIFF	arcsinh	0.2	0.01***
			INTECHAG	no	0.12	0.01**
			LOCAL	no	0.5	0.00***
			COLLECTOR	no	1.47	0.00***
			MIMEDSH	no	-0.19	0.00***
			TANGENT	arcsinh	0.23	0.01**
			AVEPRE	log	-0.2	0.00***

*Scale Parameters for Distributions of Random Parameters*						

EXPOSE (Std. Dev.) normal	0.95	0.00***	MIMEDSH (Std. Dev.) uniform		0.16	0.00***
INTPM (Std. Dev.) normal	0.53	0.00***	TANGENT (Std. Dev.) Lindley		0.46	0.00***
CPM (Std. Dev.) normal	0.10	0.00***	AVEPRE (Std. Dev.) Lindley		0.34	0.00***
HISNOW (Std. Dev.) normal	0.60	0.00***				
Dispersion Coeff	1.88		-			
Model Type	Negative Binomial		Poisson			
Log-Likelihood	-956		-937.31			
BIC	1992.72		1975.72			

(Standard errors in parentheses).

${}^{⁎⁎⁎}\;p<0.01$; ${}^{⁎⁎}\;p<0.05$; ${}^{⁎}\;p<0.1$.

The first effect, route number (ROUTE), is typically used for identification purposes but was discovered to be associated with a decrease in crashes. In Washington, the route number denotes the distance from the meridian line, implying that lower route numbers are more representative of built and urban environments. While local roads and public vehicular areas in metropolitan regions may feature more pedestrians and distractions, potentially escalating the frequency of unfocused driving and secondary task behavior among drivers [78], it is crucial to consider broader systemic factors that might contribute to the reduction of crashes along specific routes. These factors could encompass the design of the road network, the quality of infrastructure, and other elements consistent with the safe system approach to road safety. Moreover, Wu et al. [145] found that road classes are correlated with crashes, particularly in relation to their speed and urban-density characteristics. The results offered by the framework are in line with this finding, showing that the higher-speed road class categorized as collector roads (COLLECTOR) has a higher crash likelihood compared to the lower-speed road class categorized as local roads (LOCAL).

Annual average daily travel (AADT) and segment length in miles (LENGTH) were positively associated with crash-likelihood; where it has commonly been reported that the exposure effect should be modelled as a function of these two factors [1]. In addition, it is common for the logarithmic transformation to be applied to these variables [140]. Therefore, it was reassuring that the proposed hypothesis testing framework was able to identify these effects with the appropriate transformations to include in the final model.

Another significant effect included in the final model, is the number of grade breaks in the segment (GRADEBR), which is an increase in the radius of the road that results in the length of a vertical curve below its minimum length. Therefore, GRADEBRs often require drivers to slow down when faced. They are unlike speed bumps, which are purposely crafted in a road design, but are rather a natural occurrence because of terrain. Milton et al. [100] found that GRADEBRs decreased the probability of crashes and hypothesized that this counter-intuitive result was due to risk adjustment behavior when a grade break occurs. Our model hypothesized that grade breaks are positively associated with crashes; hence, these natural obstacles should be avoided when possible for the construction of new roads. The same can be stated for number of interchanges in the segment (INTECHAG), which slightly increased the probability of crashes. This result contradicts the counter-intuitive finding in Venkataraman et al. [135], where the same data was used.

Maximum median shoulder width in feet (MXMEDSH) and minimum median shoulder width in feet (MIMEDSH) were positively and negatively associated with crashes. Collectively, both MXMEDSH and MIMEDSH suggest that an increase in shoulder width, increases the likelihood of a crash, which is not an intuitive result. However, the proposed model does not feature information on the wide centre line treatments (WCLTs) and presence of Audio Tactile Lane Marking (ATLM) as they may influence the occurrence of road crashes with minimum and maximum shoulder widths. Therefore, there is potential for an analyst to revisit these variables when exploring potential mitigation strategies. MIMEDSH was also modeled with random parameters that follow a uniform distribution, which might further explain some unobserved heterogeneity across segments. This suggests that even as minimum shoulder widths increase, certain segments may become more unsafe, potentially because drivers may tend to behave more recklessly where this factor is present. Generally, shoulder widths have been found to influence driver variability with regard to positioning and speed; wider shoulders contribute to vehicles traveling at higher speeds and positioning vehicles less centrally in the lane [110]. Therefore, through MXMEDSH it was discovered that road safety actually decreases as shoulder width becomes excessive, which is further supported by Singh Bisht and Tiwari [123]. Generally, the frequency of collisions can be minimized by decreasing the maximum median shoulder width and increasing the minimum median shoulder width within road segments, suggesting that a balance between the two may be attainable. However, this does not necessarily enhance road safety in terms of fatal crashes as the crash data only accounts for the total number of crashes. While increased shoulder widths provide drivers with a recovery area, this might also encourage more reckless driving [67], [98].

A contributing factor related to curve characteristics, tangent length in the segment (TANGENT), was found to be highly significant. It was observed that as TANGENT increases, the likelihood of crash frequency also rises. This finding is in line with the results from Anastasopoulos and Mannering [10], where it was suggested that drivers tend to adopt risk-adjusting behaviors that mirror this outcome. However, a slight deviation was noted in the proposed model, where this contributing factor was more appropriately modeled with random parameters under the assumptions of the Lindley distribution to capture unobserved heterogeneity. This approach more accurately reflects the reality that in certain road segments, the number of crashes can exceed what the normal distribution would predict, particularly due to Lindley's long tail and strictly positive skew. These findings suggest that causative focus should be on risk-adopting behavior among drivers, as it does not mitigate the potential for crashes when TANGENT introduces riskier terrain, resulting in more crashes in certain segments.

The proposed model indicates that as the number of lanes in increasing milepost direction (INCLANES) increases, the probability of crashes decreases. This is likely because an increase in the number of lanes provides better opportunities for drivers to regain control, as more lanes can reduce the overall density of vehicles in individual lanes [98]. Additionally, INCLANES is transformed by a logarithm, suggesting that while an increase in INCLANES is associated with fewer crashes, the decision to increase INCLANES should be made with careful consideration. This is because the marginal benefits of increasing INCLANES diminish with each additional lane due to the properties of the logarithmic transformation.

Lastly, AVEPRE was found to decrease the probability of crashes within the mean, which is somewhat counter-intuitive. However, as it rains, drivers tend to travel at lower speeds, which could explain this outcome [69]. Unobserved heterogeneity was captured within the standard deviation and suggests an increase in crashes as rain increases, helping to explain that in more harsh conditions, the roads can become less safe. Increased rainfall, is most likely a better predictor for crash-severity and not frequency. In addition to the modeling decisions found and analyzed, the extensive hypothesis testing framework can reveal insights that an analyst might overlook. However, it does indeed require the analyst's interpretation of the results.

### 6.6 Queensland heavy vehicle data

To test the ability of the proposed framework to benefit from existing information and/or knowledge, this experiment included two distinct initialization scenarios. The first scenario, included the final model proposed by Behara et al. [21] as an initial solution or starting point to begin extensive hypothesis testing. Starting from this initial solution, the SA algorithm searched for new specifications, applying perturbations to the original model. This process led to the discovery of a new model that improved upon the original BIC, further confirming our hypothesis that an extensive search is likely to capture additional information which translates into better goodness of fit and insights.

The benchmark specification proposed by Behara et al. [21] was included in Table 5^8^ and compared to the specification discovered by the proposed framework. By utilizing prior knowledge, the proposed framework benefits by considering factors already deemed as contributing to crashes, but also uses that information to search for additional alternatives.

**Table 5:** Specification found by Behara compared against the proposed framework.

Benchmark				Estimated specification by the proposed extensive hypothesis testing using SA			
Effect	*τ*	Coeff	Pr |*z*| > *Z*	Effect	*τ*	Coeff	Pr |*z*| > *Z*
CONSTANT	no	-7.44	0.00***	CONSTANT	no	-5.49	0.00***
MCV	log	1.59	0.00***	MCV	log	1.72	0.00***
RSMS	no	-14.60	0.00***	RSMS	no	-17.36	0.00***
US	no	0.57	0.21	FW	sqrt	-0.93	0.03**
AADT	log	0.49	0.18	AADT	sqrt	0.56	0.04**
Curve50	no	-0.87	0.08*	FW RS	no	0.68	0.08*
RSHS	no	2.46	0.00***	VS Curve	no	-17.29	0.00***

*Scale Parameters for Distributions of Random Parameters*							

AADT (Std. Dev.) normal		0.10	0.82	VS Curve (Std. Dev.) normal		0.90	0.07*
Curve50 (Std. Dev.) normal		0.50	0.32				
RSHS (Std. Dev.) normal		0.07	0.90				
Dispersion Coef	1.92				1.87		
Model Type	Negative Binomial				Negative Binomial		
Log-Likelihood	-200.27				-198.24		
BIC	484.83				463.16		

(Standard errors in parentheses).

${}^{⁎⁎⁎}\;p<0.01$; ${}^{⁎⁎}\;p<0.05$; ${}^{⁎}\;p<0.1$.

The considerable improvement in BIC of the model generated by the proposed framework, compared to the original study, highlights that the analysts, despite all their knowledge and experience, were not able to escape local optima as illustrated in Fig. 6b. The convergence plot in Fig. 6b underscored the initial exploration performed by SA considering inferior solutions, before converging on an improved alternative. Numerous alternative specifications, derived from the initial solution, were subsequently tested, leading to the discovery of new models that were superior in terms of BIC and insights. The enhancement in BIC was largely attributed to an improved log-likelihood and a reduction in the number of model parameters, which further support that the model is not over-fitted [137]. The final proposed model further substantiated the assertion that the quality of model estimation can be augmented through a more strategic selection of contributing factors and explicit consideration of potential heterogeneity, as suggested by Lord et al. [92]. The newly developed model, resulting from the extensive hypothesis testing, is presented in Table 5.

The final model, refined through a extensive hypothesis testing framework, is shown in Table 5. The model demonstrates a decrease in the BIC value from 484 to 463. This improvement is primarily due to the inclusion of new factors: formation width (FW), formation width on rural single carriageways (FW RS), and very sharp curvature (VS Curve). Conversely, urban single carriageways (US), significant curvature (moderate to very sharp which is longer than 50% of the segment) (Curve50), and rural single and high speed (RSHS) were removed from the model. The proposed model incorporates a square root transformation of AADT, potentially offering a more accurate representation of its effect. This transformation facilitated the elimination of a random parameter, thereby improving the BIC and helping to avoid overfitting.

A key insight from this model is the role of FW, which encompasses the length of the carriageway and the shoulders. Typically, FW is associated with increased road safety, particularly in terms of reducing fatal crashes. This is because an increase in the number of lanes (Nlanes), and increase in lane width in conjunction with wider shoulders, offers drivers more space for maneuvering and regaining control [98], [110]. However, the model implies that this is not the case for FW RS, which are single-lane roads where the formation width is comprised solely of shoulder width and lane width. The current state of these rural single-lane carriageways suggests that their formation width might be excessively large, potentially due to wide shoulders [123] or wide lanes [81]. This excessive width in either situation could encourage riskier driver behavior and decreased driver attention. This potentially leads to an increase in fatal crashes, particularly considering the absence of additional lanes, which restricts mobility [99], [146].

Interestingly, VS Curve was identified as a significant factor in reducing crash frequency. This finding might seem counterintuitive, given the common perception that sharp curves increase crash risk—a notion supported by the benchmark model through Curve50 and affirmed by research literature [21], [53], [149]. However, contrary to these expectations, it was hypothesized that highways with notably sharp curvature likely benefit from risk-adjusting behaviors, such as drivers reducing their speed [106]. Additionally, effective mitigation strategies already implemented on Queensland highways appear to be enhancing safety. This reduction in accidents is likely attributable to various safety countermeasures, including enhanced signage and delineation, high-friction pavements, and wider clear zones Antonucci et al. [12], Gooch et al. [53]. Considering the historically common occurrence of accidents on sharply curved highways [105], and the currently low frequency of crashes in the Queensland data on sharply curved highways, it suggests that concerns regarding road design have been effectively addressed. However, it is important to note that while the model suggests improved safety from sharp curves within the mean, the associated random parameters account for the heterogeneity of these road segments. This indicates an increase in crashes within certain segments from the mean, particularly those characterized by sharp curvature. The second scenario in this experiment represents a more restrictive case, relative to the first scenario, when no model nor expert knowledge is available to provide an initial solution. This scenario represents the worse and most challenging case for the proposed extensive hypothesis framework. It could reflect cases when the analyst team has very limited crash and statistical analysis knowledge and experience. Results from this scenario are provided in Appendix A.1. As expected, the model estimated under this scenario, although comparative to Behara et al. [21], is not as fitted as the one under the previous scenario and included in Table 6.

## 7 Conclusion

This study introduced a mathematical programming formulation and optimization framework to support the estimation of data count models by facilitating extensive hypothesis testing. This includes simultaneous consideration of potential contributing factors, random parameters and their corresponding distributions, data transformations, and the selection of probabilistic models. The proposed framework offers several advantages, including the possibility of identifying additional patterns or insights that might otherwise be missed using conventional methods. Furthermore, the proposed framework reduces bias and saves time as optimization is used to generate specifications and evaluate numerous hypotheses, rather than traditional methods where an analyst might impose pre-existing knowledge and potentially overlook relevant modeling aspects. If desired by the analyst, constraints can be used to include knowledge, experience, and preferences, providing flexibility and the capability to utilize any conventional method in a much more efficient manner. This was specifically demonstrated using data from Queensland, where an initial solution was proposed at the start of the search and found to provide more evidence for superior models.

Three metaheuristics were implemented and tested to solve the proposed mathematical program for the estimation of data count models. The experiments utilized different datasets with distinct characteristics, and the results illustrated the effectiveness of metaheuristics in supporting the estimation of advanced statistical models for crash data analysis. Notably, the proposed framework successfully captured the underlying data generation process across all experiments, outperforming both published models and synthetic data. The proposed framework for crash data count model estimation was able to improve the BIC beyond what published models achieved. Moreover, the effectiveness of the proposed framework was evaluated through an experiment using synthetic data. These findings together provide strong evidence of the framework's ability to analyze crash data and capture the underlying data count generation process.

In the experiment using data from Washington state, it was discovered that found that shoulder widths exceeding a certain threshold, specifically higher than the average of all shoulder width values in the dataset, is likely to compromise safety. Additionally, it was found that speed, interchanges and grade breaks all increased crash frequency. On the other hand, number of lanes decreased crash frequency. Furthermore, the utilization of a mixture of random parameters with varying distributions across contributing factors offers superior estimation in comparison to employing a single distribution for all contributing factors. This allows for a better understanding of the heterogeneity in the data and providing a more robust estimation of parameters. In an experiment using data from Queensland, the integration of an initial solution into the search process demonstrated the ability and value of using analyst knowledge. This experiment also revealed the propensity of traditional analyst methods to get trapped in local optima. Incorporating knowledge into the proposed framework led to several findings. It was discovered that roads with very sharp curvature already have effective mitigation measures in place, suggesting that Queensland roads should be commended for their effective safety precautions. The frequency of crashes was also found to decrease with the expansion of formation width. However, a deviation was observed on rural single roads, where an increase in the formation width actually led to a higher frequency of crashes.

Both experiments using real data show the ability of the proposed framework for solving data count problems for rare events and capable of handling dispersion. Furthermore, these experiments illustrated significant time savings because all models were estimated without significant human intervention beyond verification and the corresponding analysis. With respect to the published benchmark models, the proposed framework shared many similar aspects, suggesting that the extensive hypothesis testing framework can be used with other datasets to help find appropriate specifications.

All estimated models are easily interpretive; a machine learning model may provide better statistical fits, but interpretability is a challenge. When discovering the causality of crash outcomes, the potential of metaheuristics to support the estimation of data count models is emphasized. The models found with the help of the simulated annealing algorithm did not take much time and effort to estimate while outperforming the corresponding results in Behara et al. [21] and Washington et al. [140]. Furthermore, recapturing the synthetic model with the objective of the minimization of the Bayesian Information Criterion, confirms that our objective function is sound for this single-objective minimization problem, Extensions to improve the proposed framework can be implemented by addressing some of limitations. Multiple objectives can allow an analyst to balance trade-offs between goodness-of-fit and prediction. For example; while the Bayesian Information Criterion is efficient in avoiding overfitting issues due to its penalization of the number of parameters in the model [137], a validation metric such as mean absolute error will specifically target overfitting and ensure that the model can adjust to new data [141]. Other objectives could be considered, for example; the causality of the effects on the count outcome may be of more use when the desired number of modelling effects is required. Considering the number of parameters as an additional objective can provide flexibility in the proposed extensive hypothesis testing framework. In addition to the multiple objectives, considerations regarding alternative solution algorithms cannot be overlooked. While SA was superior for the experiments performed in this study, DE and HS are more suited to multi-objective problems, and other hybridized/memetic algorithms could be considered [39]. The exploration of multiple objectives and enhancement to the solution algorithms are proposed for future research.

## CRediT authorship contribution statement

**Zeke Ahern:** Conceptualization, Data curation, Formal analysis, Investigation, Methodology, Resources, Software, Writing – original draft, Writing – review & editing. **Paul Corry:** Methodology, Supervision, Writing – review & editing. **Wahi Rabbani:** Supervision. **Alexander Paz:** Conceptualization, Methodology, Supervision, Validation, Writing – review & editing.

## Declaration of Competing Interest

The authors declare that they have no known competing financial interests or personal relationships that could have appeared to influence the work reported in this paper.

## Figures and Tables

**Table 6:** Specification found by Behara compared against the proposed framework, achieved without an initial solution.

Benchmark				Estimated specification by the proposed extensive hypothesis testing using SA			
Effect	*τ*	Coeff	Pr |*z*| > *Z*	Effect	*τ*	Coeff	Pr |*z*| > *Z*
CONSTANT	no	-7.44	0.00***	CONSTANT	no	-37.48	0.00***
MCV	log	1.59	0.00***	LENKM^*a*^	no	0.01	0.00***
RSMS	no	-14.60	0.00***	MtCurve	no	0.03	0.01**
US	no	0.57	0.21	C50:RT	no	-0.61	0.00**
AADT	log	0.49	0.18	MtTer	no	0.33	0.00***
Curve50	no	-0.87	0.08*	LW:RS	arcsinh	-1.43	0.00***
RSHS	no	2.46	0.00***	LW:US	arcsinh	4.76	0.00***
				US	no	21.17	0.00***
				RSMS	no	26.27	0.00***
				RD	no	1.35	0.00***
				RSHS	no	-19.21	0.00***
				RSLS	no	-18.36	0.00***
				AADT	arcsinh	1.02	0.00***
				HSP	no	20.94	0.00***
				SW	arcsinh	-0.05	0.00***
				FW:US	arcsinh	-2.48	0.00***
				ATLM	no	0.89	0.00***


Benchmark				Estimated specification by the proposed extensive hypothesis testing using SA			
Effect	*τ*	Coeff	Pr |*z*| > *Z*	Effect	*τ*	Coeff	Pr |*z*| > *Z*
*Scale Parameters for Distributions of Random Parameters*							

AADT (Std. Dev.) normal		0.10	0.82	AADT (Std. Dev.) normal		0.05	0.00***
Curve50 (Std. Dev.) normal		0.50	0.32	HSP (Std. Dev.) normal		0.35	0.00***
RSHS (Std. Dev.) normal		0.07	0.90				
Dispersion Coef	1.92			Dispersion Coef	1.01		
Model Type	Negative Binomial			Model Type	Negative Binomial		
Log-Likelihood	-200.27			Log-Likelihood	-171.16		
BIC	484.83			BIC	482.20		

^*a*^See Appendix A.3 for dictionary definitions.

(Standard errors in parentheses).

${}^{⁎⁎⁎}\;p<0.01$; ${}^{⁎⁎}\;p<0.05$; ${}^{⁎}\;p<0.1$.

**Table 7:** Notation: Representation of the solution and the properties of metaheuristics.

Experimental Default Parameters	

*MaxTime*	Max allowable time for each algorithm (10000 seconds of CPU time).
*H*_draws_	Number of Halton draws for sampling from the respective distributions.

Solution Representation	

*v*_*p*_	Solution vector to the mathematical program, where *p* depicts an indexed solution from the population of solutions.
*v*_*p*,*j*_	Specification of decision element k∀k∈K.
*ℓ*_*p*,*j*_	Function to determine the index value of the solution *s* for decision element *j*.

Harmony Search	

*PAI*	Pitch adjustment index.
*PAR*	Pitch adjustment rate.
*HM*	Set of distinct solutions (*v*_*s*_) in the harmony memory.
*HMS*	Harmony memory size (controls the set size of *HM*).
*HMCR*	The probability of retaining the previous solution.

Differential Evolution	

*ℓ*_*p*,*j*_	Function to determine the index of the current decision of population element p for element j.
*CR*	The probability of a crossover occurring on specification *v*_*p*,*j*_
*PS*	The population size.
*PAI*	Adjustment index.
*POP*	Set of distinct solutions (*v*_*s*_) in the population (equivalently *HM*).

Simulated Annealing	

*T*_*s*_	Number of iterations before the temperature is reduced.
*T*_*α*_	Temperature reduction factor.
*χ*_*n*_	Desired number of initial solutions used to calculate the initial temperature.
*χ*	Desired proportion to accept a solution at the beginning of the search, as a result of calculating the initial temperature

## Data Availability

The data that support the findings of this study are available from the corresponding author (Zeke Ahern, zeke.ahern@hdr.qut.edu.au) upon reasonable request.

## Appendix A

### A.3 Acronyms

**AADT** annual average daily travel.

**AIC** Akaike's Information Criterion.

**ATLM** presence of Audio Tactile Lane Marking.

**AVEPRE** average precipitation of rainfall.

**BIC** Bayesian Information Criterion.

**C50** significant curvature (longer than 50% of the segment).

**COLLECTOR** collector roads.

**COM-Poisson** Conway-Maxwell-Poisson.

**CR** crossover rate.

**Curve50** significant curvature (moderate to very sharp which is longer than 50% of the segment).

**DE** Differential Evolution.

**FW** formation width.

**FW RS** formation width on rural single carriageways.

**GA** Genetic Algorithm.

**GP** Generalized Poisson.

**GRADEBR** number of grade breaks in the segment.

**HM** harmony memory.

**HMCR** harmony memory consideration rate.

**HMS** harmony memory size.

**HS** Harmony Search.

**HSP** high-speed limit > 100 km/hr.

**INCLANES** number of lanes in increasing milepost direction.

**INTECHAG** number of interchanges in the segment.

**LEN YR** segment length multiplied by period of analysis.

**LENGTH** segment length in miles.

**LENKM** length in km.

**LOCAL** local roads.

**LW** lane width.

**MIMEDSH** minimum median shoulder width in feet.

**ML** machine learning.

**MSL** maximum simulated log-likelihood.

**MtCurve** mountainous terrain with significant curvature.

**MtTer** mountainous terrain.

**MXMEDSH** maximum median shoulder width in feet.

**NB** Negative Binomial.

**Nlanes** number of lanes.

**NN** neural network.

**PAI** pitch-adjustment index.

**PAP** pitch-adjustment proportion.

**PAR** pitch-adjustment rate.

**PS** population size.

**PSO** Particle Swarm Optimization.

**RD** rural dual carriageway.

**RF** random forrest.

**ROUTE** route number.

**RS** rural single carriageways.

**RSHS** rural single and high speed.

**RSLS** rural single and low speed.

**RSMS** rural single and medium speed.

**RT** rolling terrain.

**SA** Simulated Annealing.

**SPF** safety performance function.

**SVM** support vector machine.

**SW** shoulder width.

**TANGENT** tangent length in the segment.

**US** urban single carriageways.

**VS Curve** very sharp curvature.

**WCLTs** wide centre line treatments.

**ZI** Zero-Inflated.

## Footnotes

2 Note that all of these steps constitute only one iteration of the metaheuristic algorithm.

## Footnotes

1 This is also referred to as the offspring function.

3 Metacountregressor was developed by Zeke Ahern: The principal investigator of this paper.

4 For a complete formulation of the Poisson distribution see Lord et al. [91].

5 For a complete formulation of the NB distribution, see Mannering et al. [97].

6 For a complete formulation of the GP distribution, see Famoye et al. [43].

7 The offset effect of the model was analyzed in the same way for fair comparison: offset = segment length multiplied by period of analysis (LEN YR) * 1000) / ln(10).

8 The model in the paper was re-estimated to compute MSL to facilitate comparison.
